# Patterns of foot complaints in systemic lupus erythematosus: a cross sectional survey

**DOI:** 10.1186/s13047-016-0143-8

**Published:** 2016-03-22

**Authors:** Simon J. Otter, Sunil Kumar, Peter Gow, Nicola Dalbeth, Michael Corkill, Maheswaran Rohan, Kevin A. Davies, Sam Pankathelam, Keith Rome

**Affiliations:** Health and Research Rehabilitation Institute and School of Podiatry, AUT University, Auckland, New Zealand; Rheumatology Department, Counties Manukau District Health Board, Auckland, New Zealand; Department of Rheumatology, Auckland District Health Board and Faculty of Medical and Health Sciences, The University of Auckland, Auckland, New Zealand; Rheumatology Department, Waitemata District Health Board, Auckland, New Zealand; Biostatistics Department, AUT University, Auckland, New Zealand; Rheumatology Department, Brighton and Sussex Medical School, Brighton, UK; Rheumatology Department, East Sussex Healthcare Trust, Eastbourne, UK; School of Health Science, University of Brighton, 49 Darley Rd, Eastbourne, BN20 7UR UK

**Keywords:** Systemic lupus erythematosus, Foot, Pain, Disability, Impairment, Quality of life

## Abstract

**Background:**

Foot complaints are common in inflammatory arthropathies such as rheumatoid arthritis and cause considerable disability. However, little is published about the nature and extent of foot complaints in systemic lupus erythematosus (SLE). We aimed to explore foot complaints among people with (SLE) and to evaluate the associations between foot pain and self-reported activities of daily living and well-being.

**Methods:**

We developed and tested a new 40-item item self-administered questionnaire, using a five-stage development process utilising patient involvement throughout to ensure face and content validity. The self-administered instrument was posted to 406 people with SLE attending adult rheumatology clinics across three health boards in Auckland, New Zealand. The questionnaire enquired about symptoms of foot pain, extra-articular features, anatomical distribution of symptoms according to validated foot-mannequins and the impact of foot symptoms on activities of daily living and well-being.

**Results:**

In total, 406 questionnaires were posted, with 131 responses (response rate 32 %). We found 89 % were women, mean (SD) age 51 (15) years, mean (SD) diagnosis 12.5 (11.1) years. Overall, 77 % of those responding to the questionnaire reported foot pain during their SLE, with 45 % reporting current foot pain. All regions of the feet were affected, with the hindfoot (32 %) and ankles (30 %) most troublesome. The most common self-reported extra-articular foot complaints were cold feet, swelling and numbness. Almost two-thirds (61 %) reported foot pain adversely affected their lives; foot pain prevented sleeping in 36 % and had a negative effect on emotions for 33 %. Only 33 % of participants had seen a podiatrist. Significant association was found between foot pain and standing longer than 15 min (*p* < 0.001), walking (*p* < 0.001), climbing stairs (*p* < 0.001) and going shopping (*p* < 0.001). Pain was the primary symptom to affect quality of life (47/100).

**Conclusion:**

Foot complaints in SLE are heterogeneous in nature, and may have a substantial negative impact on patient well-being. Foot complaints need to be addressed to reduce the burden of SLE and our findings support the need for wider access to specific foot care services.

**Electronic supplementary material:**

The online version of this article (doi:10.1186/s13047-016-0143-8) contains supplementary material, which is available to authorized users.

## Background

Systemic lupus erythematosus (SLE) is a chronic, systemic, autoimmune disease that can lead to substantial multi-organ pathology [1, 2]. SLE is clinically heterogeneous; typically with a relapsing and remitting course having a negative effect on health, quality of life, career development and raising a family [3–5]. SLE is more prevalent among those with an African, Asian or Polynesian ancestry [1, 6]. There remain considerable unmet medical needs for people with SLE [4]; yet the prevalence of foot complaints in SLE is reportedly high, with 67 % of SLE participants having arthropathy in the feet [7]. A recent ultrasound imaging study found greater foot involvement than hand involvement in SLE with 73 % of participants presenting with inflammatory foot joint abnormalities [8]. These included; joint effusion, synovial hypertrophy and positive power Doppler signals [8]. Furthermore, patients with SLE are reported to be at greater risk of reporting complications due to vascular pathology secondary to accelerated atherosclerosis [9, 10]. Previous studies have found peripheral vascular disease (PVD) to be widespread among people with SLE, with a prevalence rate between 13 and 28 % [11, 12]. In the lower limb, low ankle brachial pressure indices, a key indicator of PVD, were found in 37 % of a UK study [13] and 21 % in a Swedish cohort [14]. Critical ischaemia, foot ulceration, digital gangrene, and Raynaud’s phenomenon have been reported in two large retrospective studies [15, 16]. Despite the range of reported pathologies affecting the feet, there remains a lack of evidence defining the pattern of foot involvement on function and quality of life in people with SLE. Furthermore, a recent narrative review of foot complaints in SLE also reported there is limited evidence on the epidemiology or treatment recommendations for foot disease in SLE [17]. We aimed to explore self-reported foot complaints among people with SLE and to evaluate associations between reported foot pain and clinical characteristics, specifically activities of daily living well-being.

## Method

### Subjects and setting

Participants were identified using Auckland, Counties Manukau and Waitemata District Health Board databases in Auckland, New Zealand. The Systemic Lupus International Collaborating Clinics (SLICC) classification criteria [18] were not used to identify potential participants, as this criteria is not used locally for diagnosis. Therefore, participants eligible for the study were >18 years old, had a positive diagnosis of SLE as determined by their consultant rheumatologist and had attended a rheumatology clinic for their SLE in the previous 2 years. Analysis of the rheumatology database enabled us to exclude those with juvenile SLE and other concomitant inflammatory arthropathies. Participants with undifferentiated connective tissue disorders and overlap syndromes were also excluded. Ethical approval was granted by Auckland University of Technology Ethics Committee.

### Data generation

Data were generated from a questionnaire (Additional file 1) designed to collect information relating to demographic and clinical characteristics’ that included: age, sex, body mass index (BMI), ethnicity, employment status, current medications, smoking status, SLE disease duration and morning stiffness. Details of questionnaire development process are provided in Additional file 2 with data from our pilot study in Additional file 3. The anatomical location of foot pain using three different case definitions (currently, during the past month and ever during the course of SLE) were recorded with the use of validated foot mannequins [19]. The severity of foot pain was assessed with using a 10 cm Visual Analogue Scale (VAS). The self-reported presence of vascular, neurological and cutaneous extra-articular features complaints affecting the feet was also recorded. The effect of foot complaints on participants’ well-being was captured by enquiring about sleep and emotion. The impact of foot symptoms on activities of daily living (walking, shopping, climbing stairs, wearing different shoes) as well as family and social activities were reported. The assessment foot pain, need for foot care and any foot-specific treatment received was also recorded. We also used a SLE quality of life questionnaire (the Lupus QoL) a 34-item questionnaire across eight domains (physical health, pain, planning, intimate relationships, burden to others, emotional health, body image and fatigue) [20]. Scores are computed to calculate a total score from 0 (worst quality of life) to 100 (best quality of life) for each domain [20].

### Data analysis

Data were entered into SPSS version 22 (IBM Inc., Armonk, New York, USA). Sex, ethnicity, work status, smoking status and clinical characteristics of disease duration, current pharmacological management, the location of foot pain, and extra articular foot complaints and the impact of foot complaints on activities of daily living and the assessment/management of foot complaints were reported as number (percentage). Other demographic characteristics (age and BMI) together with clinical characteristics of disease duration, early morning stiffness and the severity of foot pain were reported as mean (SD). Chi-square was used to determine associations between the presence of foot pain and categorical variables of foot-related activities of daily living (standing, walking, climbing stairs, going shopping and wearing different shoes). A level of significance was set at the 5 % level. Recommendations of the STROBE group [21] were applied when reporting the findings.

## Results

In total, 406 questionnaires were posted with 131 responses (response rate 32 %). The demographic and clinical characteristics are summarized in Table 1. Participants were predominantly female (*n* = 117, 89 %), with mean (SD) age 51 (15) years old and a mean (SD) disease duration of 12.5 (11.1) years. The majority of participants were paid workers (*n* = 68, 52 %) and of New Zealand European ethnicity (*n* = 67, 51 %).

**Table 1:** Demographic and clinical characteristics

Characteristic	Value
Sex, *n* (%)	Female: 117 (89)
	Male: 14 (11)
Age (years), mean (SD)	51 (15)
Disease duration, mean (SD)	12.5 (11)
BMI (Kg/m^2^), mean (SD)	27.7 (6.4)
Current smoker, *n* (%)	27 (21)
Employment status, *n* (%)	
Paid work	68 (52)
Not working	32 (24)
Retired	20 (15)
Unpaid work	5 (4)
Sick leave	2 (2)
Full-time education	3 (2)
Not stated	1 (1)
Ethnicity, *n* (%)	
New Zealand European	71 (54)
Pacific Island	23 (18)
Asian	24 (19)
Māori	11 (8)
Early morning stiffness, *n* (%)	96 (68)
Early morning stiffness, duration, hours, mean (SD)	2.1 (5.4)
Hydroxychloroquine use, *n* (%)	97 (74)
Azathioprine use, *n* (%)	25 (19)
Methotrexate use, *n* (%)	19 (14)
Mycophenolate use, *n* (%)	6 (5)
Cyclophosphamide use, *n* (%)	3 (2)
Oral glucocorticoids use, *n* (%)	55 (42)
Non-steroidal anti-inflammatory drug use, *n* (%)	29 (22)
Rituximab use, *n* (%)	5 (4)

We found 77 % (*n* = 99) of participants in this study reported foot pain during the course of their disease, with 52 % (*n* = 68) reporting pain in the last month and 45 % (*n* = 59) reporting current foot pain. For those participants who reported current foot pain, the mean (SD) VAS score was 4.9 (2.2) cm. No differences were found between foot pain and age (*p* = 0.72), duration of SLE (*p* = 0.08), BMI (*p* = 0.18), smoking (*p* = 0.15) or ethnicity (*p* = 0.37).

**Fig. 1:**
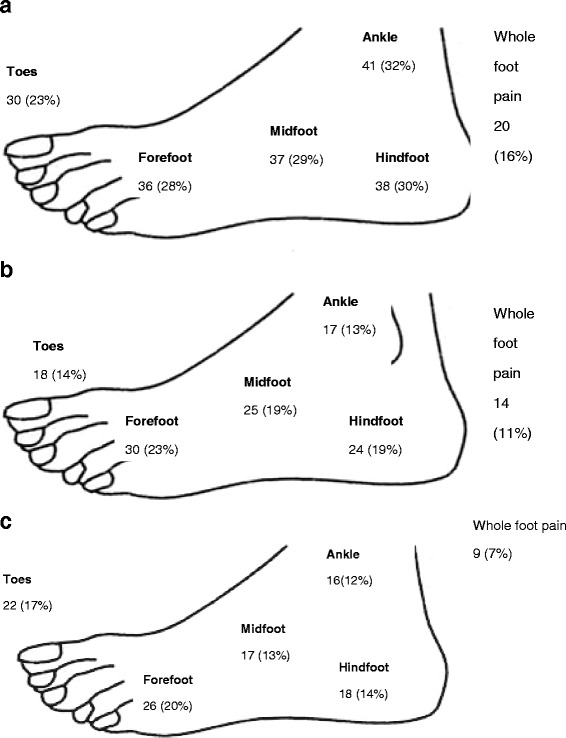
Frequency of pain reported by patients within different time descriptors at different anatomical sites in the foot. **a** Frequency of pain reported ever during the course of the disease at different anatomical sites in the foot. **b** Frequency of pain reported in the last month at different anatomical sites in the foot. **c** Frequency of pain reported today at different anatomical sites in the foot

All parts of the foot were affected during the course of the disease in our group, but overall, ankle (*n* = 40, 32 %) and hind foot pain (*n* = 37, 30 %) predominated. However, in those participants reporting current foot pain; digital (*n* = 22, 17 %) and forefoot pain (*n* = 26, 20 %) was more common than pain in the hind foot (Fig. 1). We found 91 % of participant’s self-reported joint pain as part of the initial presentation of their SLE. While 35 % (*n* = 46) reported their hands were affected initially, in contrast only 4 % (*n* = 5) reported pain in the feet as the first reported symptom. During the course of the disease, pain in the foot joints (*n* = 91, 71 %), pain in the arches of the feet (*n* = 71, 55 %) or pain in tendons (*n* = 63, 49 %) was reported by our participants (Table 2).

**Table 2:** Self-reported foot complaints in SLE (*n*, %)

Symptom reported	Always	Sometimes	Never	No response
Cold feet	57 (44)	57 (44)	11 (9)	4 (3)
Chilblains	8 (6)	37 (29)	72 (56)	12 (9)
Raynaud’s phenomenon	26 (20)	50 (38)	49 (38)	4 (3)
Intermittent claudication	16 (12)	54 (42)	57 (44)	2 (2)
Skin rash (legs or feet)	17 (13)	32 (25)	76 (59)	4 (3)
Blistering skin rash	7 (5)	19 (15)	98 (76)	5 (4)
Foot ulceration	1 (1)	18 (14)	106 (82)	5 (4)
Numbness	18 (14)	60 (47)	48 (37)	3 (2)
Loss of balance	6 (5)	42 (33)	77 (60)	4 (3)
Swelling	25 (19)	55 (43)	46 (36)	3 (2)
Joint pain	28 (22)	63 (49)	33 (26)	5 (4)
Arch pain	19 (15)	52 (40)	55 (43)	3 (2)
Tendon pain	17 (13)	46 (36)	63 (49)	3 (2)
Daily living activity				
Standing >15 min	21 (16)	48 (38)	49 (38)	11 (9)
Walking	22 (17)	49 (38)	49 (38)	8 (6)
Climbing stairs	28 (22)	33 (26)	57 (56)	10 (8)
Wearing different shoes	24 (19)	42 (33)	54 (42)	8 (6)
Shopping	23 (18)	39 (31)	58 (45	8 (6)
Foot pain: family/social activities				
Foot pain: social activities	38 (29)	51 (39)	41 (31)	1 (1)
Foot pain: family activities	26 (20)	49 (37)	54 (41)	2 (2)

Respondents reported a wide range of extra-articular complaints affecting their feet–summarised in Table 2. The most common complaints were associated with vascular changes. However, some features suggesting neurological deficit were reported. Cutaneous features that included skin rashes on the feet/legs, blisters and foot ulcers were not reported to be pathology in the majority of cases.

Quality of life in general was adversely affected by foot pain for 61 % (*n* = 78) of participants. Table 2 illustrates that for many participants difficulties were reported with standing for more than 15 min, walking, climbing stairs, wearing different shoes and to a lesser extent going shopping. Significant associations were found between foot pain and standing longer than 15 min (*p* < 0.001), walking (*p* < 0.001), climbing stairs (*p* < 0.001) and going shopping (*p* < 0.001). Additionally, respondents reported foot pain prevented sleep for 36 % (*n* = 47) of participants and 33 % (*n* = 43) reported foot pain had a negative effect on their emotions. The extent to which foot complaints affected either family or social activities varied considerably (Table 2). Pain was the primary symptom to affect overall quality of life and received the lowest score (Table 3). The domain with the highest score for quality of life was emotional health.

**Table 3:** Self-reported Lupus quality of life scores

Domain	Score
Physical health (8 items)	67
Pain (3 items)	47
Planning (3 items)	74
Intimate relationship (2 items)	71
Burden to others (3 items)	69
Emotional health (6 items)	75
Body image (5 items)	57
Fatigue (4 items)	62

In total, of 50 % (*n* = 66) of our respondents reported that they had discussed their foot complaints with their general practitioner and 49 % (*n* = 64) with their rheumatologist. When asked to recall the most recent examination of their feet, respondents reported no difference (*p* = 0.31) in the recalled time since foot examination compared with the time since hand examination. Overall, 41 respondents (31 %) indicated they had difficulty undertaking basic foot care, e.g. cutting toenails. Only 43 respondents (33 %) reported ever having seen a podiatrist. Twelve respondents (9 %) has seen a foot surgeon and 8 (6 %) undergone surgery. Overall, 29 respondents (22 %) had been prescribed insoles with 14 respondents (11 %) currently using foot orthoses and a further 15 respondents reporting they had stopped using insoles (12 %).

## Discussion

The findings from this study have shown that 77 % of participants who responded to the questionnaire reported as having experienced foot pain at some point during the course of their SLE and 45 % reported experiencing foot pain currently. Previous studies have reported articular involvement to be the most common symptom in SLE with prevalence rates between 83 and 95 % [22, 23]. Overall, 71 % of our participants reported pain in their foot joints. However, clinical validation of this finding would be appropriate given both the sampling frame used in this study and the relative close proximity of anatomical structures in the foot. When noting the location of their foot pain, more of our participants reported rearfoot pain, in contrast to a similar study in rheumatoid arthritis where forefoot pain predominated [24]. Lagnocco et al. [8] reported a high prevalence of forefoot pathology using ultrasonography, but their work did not include hind foot joints. The self-reported occurrence of foot pain in our study was higher than in some clinically based studies of inflammatory arthritis where soft tissue involvement is common [25, 26]. The impact of foot pain on foot-related activities of daily living, such as walking, were substantial in those responding to the questionnaire. It is possible that high levels of foot pain may also contribute to the considerable employment disability that has been previously been reported with SLE [27]. Mancuso and colleagues [28] indicated that for people with SLE, walking was the preferred physical activity to improve their symptoms. Yet in our sample, walking was adversely affected for over half of respondents. Notably, foot pain was also reported to negatively impact on social and family activities as well as sleep and emotional health for substantial proportion of those responding to the questionnaire, but did not reach statistical significance. In parallel, results from the Lupus Quality of Life questionnaire also indicated that increased general pain resulted in low quality of life scores.

We found foot complaints likely to be caused by impaired vasculature were commonly reported by our participants and included cold feet, Reynaud’s phenomenon and chilblains. Bhatt et al. [11] indicated 30 % of their subjects had Raynaud’s phenomenon, with 22 % previously reported by Font et al. [22]. This is broadly in line with our findings where 20 % reported Raynaud’s phenomenon was always present and sometimes present for 38 %. It remains unclear if numbness, which was a commonly reported compliant, is frank clinical neuropathy. Previous studies have suggested a prevalence of peripheral neuropathy between 6 and 14 % in SLE using retrospective studies, with not all of these cases being directly attributed to SLE [29, 30].

Cutaneous lesions are common in SLE and are reportedly the second most frequent finding after musculoskeletal symptoms [31] and muco-cutaneous lesions comprise four of the 11 items of the revised SLICC criteria [18]. However, we found self-reported cutaneous lesions on the feet were comparatively uncommon. Chilblains were the most common cutaneous complaint, reported by up to 35 %, slightly greater than the 21 % reported in a UK study [32]. The relative infrequency of cutaneous complaints may be because many of the cutaneous features seen in SLE are typically noted on the face and are photosensitive in nature [33]. It remains unclear whether those skin pathologies reported on the feet (rash, blisters and chilblains) were SLE-specific or SLE-non-specific. Finally, foot ulceration, while comparatively uncommon, was reported to have been sometimes present by 14 %, a similar proportion noted in a study on rheumatoid arthritis [34].

In spite of the frequency of reporting of foot complaints and the difficulties associated with undertaking basic foot care for some, comparatively few respondents indicated they had seen a podiatrist. Clinical guidelines [35, 36] recommend podiatric input be provided, particularly for the combination of foot pain and extra-articular features that make people with SLE particularly at risk of foot complications. Comparatively few participants had been provided with insoles, and a systematic review has demonstrated some benefit from this type of intervention in other inflammatory arthropathies, such as rheumatoid arthritis [37].

Our findings need to be considered alongside a number of limitations. Our response rate of 32 % was lower than hoped, but in line with recent work utilising a similar methodology [38]. SLE typically affects people from ethnic minorities who may not have similar levels of health literacy and may find questionnaires more complex to complete. The questionnaire relies on self-reporting of foot complaints and as highlighted previously, further clinical validation of our findings is required as some of our findings may not only be due to SLE. Equally, self-reporting may unreliable owing to an incorrect ‘self-diagnosis’. However, a recent study reported high levels of agreement between self-rpert and clinical examination for straight foward foot complaints in people with rheumatoid arthritis [39]. It is difficult to exclude the possibility of a responder bias; i.e. participants’ with prevalent foot pain were more likely to respond to this survey. There remains some reassurance in the fact that some 55 % of respondents were not currently experiencing foot pain and almost a quarter of our respondents had never experienced foot pain, yet fully completed the questionnaire. There is currently a lack of podiatric integration within rheumatology services and an unmet need for podiatric foot care for people with inflammatory arthritis in New Zealand [40]. Therefore, access to podiatry service is not similar to equivalent services offered in the UK and around the world. New Zealand has a higher proportion of people of Maori and Pacific Island heritage than other parts of the world. These groups are known to have a higher incidence of SLE [6] and as such our findings may not be fully transferrable. The mean age in our study is higher than other cross-sectional epidemiological studies [11, 12] and it is possible we have not fully captured younger participants’ perceptions. Using a cross-sectional survey, there is inevitably considerable heterogeneity (and consequently a large number of potential confounders) in the study population, however to some extent this reflects the varied nature of SLE.

Foot complaints can have serious sequelae in people who are immuno-compromised, particularly where systemic co-morbidities known to adversely affect foot health such as poor peripheral vasculature co-exist. Future work should seek to consider how the variables that may affect foot pain are investigated together, as well as to determine the risk factors associated with foot pain in SLE. The use of alternative data capture mechanisms, (e.g. utilising social media platforms) should also be considered, given the overall typically younger age profile associated with SLE. The current study has highlighted that participants with SLE have an increased need for a range of basic foot care services. Further work is also needed to develop and validate a patient-reported outcome tool to evaluate foot pain, impairment and disability in people with SLE. This would better enable clinical trials evaluating podiatric interventions in SLE to be undertaken.

## Conclusions

We have characterised the patterns and effects of foot complaints in a group of people with SLE. Pain in the joints of the feet was more common than extra-articular features. Nevertheless, a wide range of extra articular complaints was reported from vascular, neurological and cutaneous origin. Foot complaints in SLE appear heterogeneous in nature, and may have a substantial negative impact on participants’ mobility, quality of life and well-being. There would seem to be a need for wider access to specific foot care services and the development of a patient-reported outcome tool to evaluate foot pain, impairment and disability in people with SLE would be valuable.

## Additional files

Additional file 1: Questionnaire to survey foot complaints among people with systemic lupus erythematosus. This file contains a final copy of the questionnaire posted to people with SLE. (PDF 269 kb) Additional file 2: Development of the questionnaire. This file contains a detailed report of the steps taken to develop and test the questionnaire. (DOCX 21 kb) Additional file 3: Pilot study results. This file details the findings from the pilot study used to test the questionnaire in it’s final stage of development. (DOCX 15 kb)
