# Supplementary material for: Patterns of foot complaints in systemic lupus erythematosus: a cross sectional survey
Source: J Foot Ankle Res. 2016 Mar 22;9:10. doi: 10.1186/s13047-016-0143-8 (PMC4802627; doi:10.1186/s13047-016-0143-8)
Supplement: Additional file 2: — Development of the questionnaire. This file contains a detailed report of the steps taken to develop and test the questionnaire. (DOCX 21 kb) [file 13047_2016_143_MOESM2_ESM.docx]

**Additional file 2 Development of the questionnnaire**

*Stage 1 – derivation of questionnaire content*

We designed a questionnaire from first principles with the aim of identifying, from a patient’s perspective, the nature and extent of complaints affecting the feet in those with SLE. This foot-specific questionnaire was devised by initially combining aspects from a series of different, yet complementary sources. Firstly, illness narratives [1] from people with SLE attending an out-patient podiatry clinic were gathered to gain a patient-centred understanding of their reported foot problems and how these were affecting their day-to-day lives. Disease activity indices specific to SLE [2-5] (Bombardier et al. 1992, Hay et al. 1993, Gladman et al. 1996, Rahman et al. 2001) were reviewed to identify the wider aspects of the disease that required inclusion. Notably these were not specific for foot involvement. Therefore an existing validated foot-centred outcome measure specific for rheumatoid arthritis (Leeds foot impact scale [6]) Helliwell and a questionnaire previously used to ascertain foot complaints in rheumatoid arthritis [7]; (Otter 2010) were consulted to determine possible domains for complaints reported by those with SLE. Internationally agreed guidelines on the assessment of SLE in trials [8] (strand and Chu 2011) and the management of Lupus [9,10] (Bertsias et al. 2008, Mosca et al. 2011) were consulted to identify the core domains that are currently considered important in practice. Finally, discussions with a consultant rheumatologists (KAD, SP) were undertaken to ensure key medical aspects of the disease that may impact on foot complaints had been accurately captured. Items from these sources were then triangulated to develop a draft questionnaire comprising 31items. This initial version included demographic details (age, gender, BMI, ethnicity and work status) together with key clinical information (disease duration and current pharmacological management. The anatomical location of foot pain via validated mannequins was included [11] (Chatterton). Extra-articular features were captured using Likert-type scales as were the effects of foot complaints on foot-related activities of daily living. Foot examination and treatment(s) received for foot complaints were also explored.

*Stage 2 – Review of - items for inclusion in the questionnaire*

In line with current EULAR recommendations [12] (de Wit), people with SLE (including those who were recently diagnosed and those with a long-standing diagnosis) were invited to participate in reviewing the first draft of the questionnaire design. Participants were a provided with an initial draft of the questionnaire, which they reviewed prior to a discussion with the lead author (SO), using a cognitive debriefing approach [13]. Some items were discarded as being unimportant (e.g. some aspects of prescribed medication), whereas other areas of importance to participants (e.g. the effects of foot complaints on participants’ well-being was captured by enquiring about sleep and emotion were added). Additionally, a greater emphasis was placed on the range of extra-articular features using a tabulated version of these questions. Following this process the questionnaire was re-drafted and a longer 40-item version produced. Notably, participants indicated they did not mind the additional items as it more fully represented their symptoms.

The questionnaire was designed to collect demographic and clinical information with questions relating to age, sex, BMI, ethnicity, employment status, current medications, duration of diagnosis and morning stiffness were recorded. The anatomical location of foot pain using three different case definitions (currently, during the past month and ever during the course of SLE) were recorded with the use of validated foot mannequins. Data relating to the severity of foot pain was assessed with using a 10cm Visual Analogue Scale (VAS). The presence of vascular, neurological and cutaneous extra-articular features were documented, The effects of foot complaints on participants’ well-being was captured by enquiring about sleep and emotion as well as the impact of foot complaints on family and social activities were considered. The assessment and management of foot complaints were also included. Finally the impact of foot pain on foot-related activities of daily living (walking, shopping, climbing stairs and wearing different shoes) was explored. A final question was left open for respondents to include further qualitative detail. Questions were designed to elicit ordinal, nominal, categorical and interval data, as well the affording participants the opportunity to provide more detailed responses in open questions.

*Stage 3 - Questionnaire testing and validation*

Patient participants then each completed the second draft 40- questionnaire and commented on its design, content and scoring via an approach described previously. [14,15] Denscombe 2003, Carr 2003) During this stage each question is considered for its face and content validity. This process is designed to enhance the applicability and feasibility of the questionnaire together with confirming face and content validity. At this point no items in the questionnaire were considered to be redundant, nor were additional items added. However, minor adjustments to the wording of some questions and some response options were undertaken. Individual items were placed into three broad categories; however within these categories no attempt was made to order questions in an attempt to reduce respondent exhaustion.

To ensure cross-cultural sensitivity an independent review was carried out by three consultant rheumatologists (PG, SK, ND) and a professor of podiatry (KR) who were not involved with the original development. The only change was an addition to the ethnicity category to ensure indigenous peoples were fully represented. Other minor changes to the typography and layout were made to improve the visual appearance and ease of reading of the final version.

*Stage 4 - Ethical approvals*

In line with current requirements, ethical approvals were sought for both the development of the questionnaire and data collection. Approval for the study was granted from University of Brighton (13-033.R1)**,** IRAS (06/Q1907/12), Auckland University of Technology ethics committee (AUTEC 14/56) and District Health Boards (1733, A+ 6335, RM 0980712808). Approval was also gained from the Maori research board. Following the pilot study (detailed in stage 5 below) written permission to use the Lupus QoL was obtained from the author.

*Stage 5 - Pilot study*

A pilot study of the questionnaire was carried out with SLE patients attending outpatient rheumatology appointments at two teaching hospitals over a 3-month period, each receiving a copy of the questionnaire. This sampling frame might inadvertently only select those with the more severe forms of the disease: therefore members of a SLE patient support group also agreed to receive copies of the questionnaire. A total of 40 participants received questionnaires as part of the pilot study. All participants were diagnosed with SLE by their consultant rheumatologist and were aged 18 years or over. From the pilot study a total of 26 usable responses were received (58%) and results are presented in appendix 3.

Overall, foot complaints were reported to be common in SLE. Foot pain was common and affected all parts of the foot, having a considerable impact on foot-related activities of daily living. A wide range of articular, vascular, neurological complaints was described indicating no redundancy of categories. Importantly, qualitative data did not indicate further categories were required. These pilot data also provided an opportunity for formal testing of items for internal consistency. Some items were deemed inappropriate given the qualitative or nominal nature of the response criteria. However, those with categorical or interval level data were tested using Cronbach's alpha and yielded good or very good levels of consistency. Scales to measure the extent and duration of foot symptoms (e.g. pain) indicated very high levels of consistency (α 0.936). Items used to assess the presence and frequency of extra-articular foot complaints had good levels of consistency (α 0.774). Those measures of foot-related activities of daily living also had high level of consistency (α 0.859).

From the pilot data it was noted even mild foot complaints seemed to have a considerable impact on well-being and aspects of quality of life. This led to consideration as to if foot complaints were cumulative in nature and/or whether the overall quality of life issues in Lupus were impacting on feet or vice versa It was felt the use of a quality of life instrument should be included to complement data collection. A disease specific measure (Lupus QoL) [16] McElhone,l, ) was preferred over a generic questionnaire (e.g. SF36) as these instruments can lack the sensitivity and specificity required, [17] (Elhone,l, Kuriya) and may not correlate well with other factors such as disease activity [18] Aggarwel). The Lupus QoL was selected owing to its ease of completion and good psychometric properties [16].

**References**

1 Kumagai AK, A Conceptual Framework for the Use of Illness Narratives in Medical Education Academic Medicine. 2008; 83: 653-658

2 Bombardier C, Gladman DD, Urowitz MB, Caron D, Chang CH, Derivation of the SLEDAI Arthritis & Rheumatism 1992;35:630-40

3 Gladman D, Ginzler E, Goldsmith C, Fortin P, Liang M, Urowitz M, et al. The development and initial validation of the system lupus international collaborating clinic. College of Rheumatology damage index for systemic lupus erythematosus Arthritis & Rheumatism 1996;39:363-369

4 Hays EM, Bacon PA, Gordon C, Isenberg DA, Maddison P, Snaith ML, et al. The BILAG index: a reliable and valid instrument for measuring clinical disease activity in systemic lupus erythematosus Quarterly Journal of Medicine 1993;86:447-458

5 Rahman P, Gladman DD, Urowitz MN, Hallett D, Tam DS, Early damage as measured by the SLICC/ACR damage index is a predictor of mortality in systemic lupus erythematosus Lupus 2001;10:939-946

6 Helliwell P, Reay N, Gilworth G, Redmond A, Slade A, Tennant A, Woodburn J Development of a foot impact scale for rheumatoid arthritis. Arthritis & Rheumatism. 2005; 53:418-422

7 Otter SJ, Lucas K, Springett K, Moore A, Cheek L, Young A, Davies K, WalkerBone K, Foot pain in rheumatoid arthritis prevalence and risk factors & management: an epidemiological study Clinical Rheumatology 2010;29:255-271

8 Strand V, Chu AD, Measuring outcomes in systemic lupus erythematosus clinical trials Expert review pharmacoeconomics outcomes research 2011;11:455-468

9 Bertasis G, Gordon C, Boumpas DT, Clinical trials in systemic lupus erythematosus (SLE); lessons from the past as we proceed to the future the EULAR recommendations for the management of SLE and the use of endpoints in clinical trials Lupus 2008;17:437-442

10 Mosca M, Tani C, Aringer M, Bombardieri S, Boumpas D, Cervera R, et al. Development of quality indicators to evaluate the monitoring for SLE patients in routine clinical practice Autoimmunity reviews 2011;10:383-388

11 Chatterton BD, Muller S, Thomas MJ, Menz HB, Rome K, Roddy E, Inter and intra-rater reliability of the scoring of foot drawings. J Foot Ankle Res 2013;6:44

12 de Wit MPT, Berlo SE, Aanerud GJ, Aletaha D, Bijlsma JW, Croucher L, et al. European League Against Rheumatism recommendations for the inclusion of patient representatives in scientific projects *Ann Rheum Dis* 2011;70:722–726.

13 Wild D, Grove A, Martin M, Eremenco S, McElroy S, Verjee-Lorenz A, et al.

Principles of Good Practice for the Translation and Cultural Adaptation Process for Patient-Reported Outcomes (PRO) Measures: Report of the ISPOR Task Force for Translation and Cultural Adaptation. Value in Health 2005;8:2

14 Denscombe M, 2003 The good research guide Open University Press 144-6

15 Carr A, Hewlett S, Hughes R et al. 2003 Rheumatology outcomes: the patient's perspective Journal of Rheumatology 2003;30:880-83

16 McElhone K, Castelino M, Abbott J, Bruce IN, Ahmad Y, Shelmerdine J, et al. The LupusQoL and associations with demographics and clinical measurements in patients with systemic lupus erythematosus. J Rheumatol 2010;37:2273–9.

17 Aggarwal R, Wilke CT, Pickard AS, Vats V, Mikolaitis R, Fogg L, Block JA, Jolly M, [Psychometric properties of the EuroQol-5D and Short Form-6D in patients with systemic lupus erythematosus.](http://www.ncbi.nlm.nih.gov.ezproxy.aut.ac.nz/pubmed/19369452) J Rheumatol. 2009;36:1209-16

18 Kuriya B, Gladman DD, Ibanez D, Urowitz MB. Quality of life over time in patients with systemic lupus erythematosus. Arthritis Rheum 2008;59:181–5.
