# Supplementary material for: Patterns of foot complaints in systemic lupus erythematosus: a cross sectional survey
Source: J Foot Ankle Res. 2016 Mar 22;9:10. doi: 10.1186/s13047-016-0143-8 (PMC4802627; doi:10.1186/s13047-016-0143-8)
Supplement: Additional file 3: — Pilot study results. This file details the findings from the pilot study used to test the questionnaire in it’s final stage of development. (DOCX 15 kb) [file 13047_2016_143_MOESM3_ESM.docx]

**Additional file 3: Pilot results**

Responses were received for 25 female subjects (age 25-81, mean disease duration 12 years (SD 10)). In total 92% reported their Lupus had caused foot pain, with 63% reporting current foot pain. The average current pain score was 2.67 on a 10cm VAS (SD 2.58). No significant correlations were found between foot pain and respondent’s age, duration of Lupus or BMI. In total 35% reported foot pain prevented sleeping and for 50% foot pain had a negative effect on their emotions. Overall, 72% reported that their lives in general were negatively affected by foot pain. Only 15% reported their foot complaints had never interfered with either social or family activities. Table S1 illustrates articular and extra-articular foot complaints reported by respondents.

Table S1 Articular and extra-articular foot complaints reported by respondents.

| **Foot symptom** | **Always** | **Sometimes** | **Never** |
| --- | --- | --- | --- |
| Cold feet | 36% | 64% | 0 |
| Chilblains | 4% | 23% | 63% |
| Colour changes | 23% | 50% | 27% |
| Intermittant caludication | 0% | 72% | 28% |
| Skin rash on foot/leg | 19% | 39% | 42% |
| Blisters on foot/leg | 4% | 19% | 77% |
| Foot ulceration | 0 | 15% | 85% |
| Numbness in feet | 12% | 50% | 38% |
| Numbness causing loss balance | 8.3% | 54.2% | 37.5% |
| Swelling | 4% | 70% | 26% |
| Pain in foot joints | 28% | 60% | 12% |
| Arch pain | 12% | 48% | 40% |
| Tendon pain | 8% | 56% | 36% |

Foot complaints had a considerable impact on foot-related activities of daily living (Table S2)

Table S2 Effect of foot pain on foot-related activities of daily living

|  | **Standing**  **>15 mins** | **Walking** | **Climbing stairs** | **Wearing shoes** | **Going shopping** |
| --- | --- | --- | --- | --- | --- |
| **All the time** | 12% | 23% | 23% | 23% | 19% |
| **Sometimes** | 42% | 46% | 35% | 38% | 42% |
| **Never** | 8% | 0 | 8% | 4% | 8% |
| **No reply** | 38% | 31% | 34% | 35% | 31% |

In terms of the assessment and management of foot complaints, most respondents (61%) had discussed their foot complaints with their rheumatologist (39% with family Doctor). Overall, feet were examined annually and hands 9 monthly, but these difference were not significant. In total 35% had difficult with basic foot care, and 50% of respondents had seen a podiatrist. In total, 25% had been provided with insoles but most (85%) did not have specialist footwear.
